# The House of Lords Proves Its Value

**Published:** 1919-05-31

**Authors:** 


### THE HOUSE OF LORDS PROVES ITS VALUE.

In The Hospital of April 26 we called attention to the fact that the Central Committee's Registration Bill, as amended in Standing Committee E. of the House of Commons, and engineered by the party which has for years fostered disunion in the nursing profession, amounts as it stands to a betrayal of the interests of the working nurse, and a travesty of justice so far as the great bulk of the members of the nursing profession is concerned. This Bill as it left Standing Committee E., if allowed to pass uncontested, would give the controlling influence to persons who have had little or nothing to do with the training, education, and creation of British nurses as they exist to-day. Members of Parliament are likely to demand protection for the nurses and their patients by substituting a Registration Bill which provides justice to all interests, and protection for the working nurse.

We publish to-day the official verbatim report of the debate in the House of Lords on Tuesday, when the second reading of Lord Goschen's Registration Bill to effect these objects was passed by a majority of practically three to one. Lord Buckmaster said that registration by itself is nothing but the compiling of a directory, and if the Lords wished to make registration real they must associate it with something that will establish the status and improve the position of the people who register. To secure this he suggested that when the Central Committee's Bill came up to the Lords both Lord Goschen's Bill and this Bill should go together to a Select Committee. Lord Buckmaster's suggestion was received with general approval by the House of Lords. There is good reason to hope that both Bills will be subjected to investigation by a Select Committee, for in this way full justice to all interests can be secured.

Lord Ampthill, who took up the question of State Registration twelve years ago, and conducted a Fenwick Bill through the House of Lords, having returned after four and a half years in the Army, felt that, having once put his hand to the plough, he could not abandon the cause he had espoused. We could wish, in justice to himself, that he had been made aware of the true inwardness of the procedure of the Central Committee party, for then he would have known that the lobbying of which he complained was mainly that of his present friends, and that they, as the Houses of Lords and Commons should know by this time, seem to have acted on the principle he named, that "one of the best ways of getting on in the political world is by sheer effrontery." He justified his motion to reject Lord Goschen's Bill on the grounds of expediency, principle, and because he regarded it as a thoroughly bad Bill. Lord Goschen's Bill rests on justice for the nurse, the nursing profession, and the public, so expediency is eliminated. Principle appears, in his Lordship's view, to rest on the fact that the House of Lords was committed to the Bill they formerly passed. But that Bill is impossible of acceptance if justice is to be done to all the interests concerned. As to the Goschen Bill being a thoroughly bad Bill, those who become seized with a knowledge of all the facts will realise speedily, when the two Bills pass to a Select Committee, that the contrary is the truth.

We agree with the view of the House of Lords, as expressed by Lord Buckmaster, that it would indeed have been a serious thing for their lordships to reject Lord Goschen's Bill, and to say that it is not to be considered at all. We have little doubt that when the two Bills get to the Select Committee, and all the facts are brought to the test of proof, Lord Ampthill's present view will be shown to be demonstrably ill-founded. Not only does the Bill before the House of Commons not hold the field, but we understand that Mr. Macmaster, the Chairman of Standing Committee E, has suggested that this Bill should be referred back to this Committee, for the Committee has been misled, and those with whom he had discussed the subject agreed with him that the Bill as it stands is not right. All honour, therefore, to the statesmanship and perspicacity of the House of Lords, which, by its vote, has defended the nursing profession from the grievous risks and injustices with which it was threatened.

Everybody will regret that Lord Ampthill should have attacked the *Times* for its accurate statement in a recent article that "a new spirit animates nurses and demands expression in democratic institutions." A great wave of awakening has passed over the whole nursing body in these islands in the last eighteen months especially, and the sign of the new spirit makes itself plainer every day to those who are in the closest touch with the working nurse. The *Times* has exhibited great impartiality in dealing with the question of the State Registration of Nurses, and the articles referred to have proved of real importance from their force and their just interpretation of the true inwardness of the present position. We have had the privilege of working with the *Times* for the best part of a lifetime, but we are without knowledge of the writers Lord Ampthill referred to. We can testify with assured conviction that the underlying motives of successive editors have invariably been, to uphold the principles best calculated to promote the interests and advance the causes with which the *Times* has dealt in its columns.

